# Correlation of preoperative radiological lymph node assessment with pathological nodal and tumor findings in tongue and oral floor squamous cell carcinoma

**DOI:** 10.1007/s00432-026-06490-0

**Published:** 2026-05-23

**Authors:** Jannik Ketschau, Yannik Leonhardt, Cornelius Leopold, Alex Grabenhorst, Hannes Singer, Jonathan Mohr, Nils Krautkremer, Katharina Pippich, Herbert Stimmer, Klaus-Dietrich Wolff, Lucas M. Ritschl

**Affiliations:** 1 Department of Oral and Maxillofacial Surgery, TUM University Hospital Klinikum Rechts der Isar, School of Medicine and Health, Technical University of Munich, Ismaninger Strasse 22, 81675 Munich, Germany; 2 Institute of Diagnostic and Interventional Radiology, TUM University Hospital Klinikum Rechts der Isar, School of Medicine and Health, Technical University of Munich, Munich, Germany

**Keywords:** Head and neck imaging, Oral squamous cell carcinoma, Cervical lymph node metastasis, Radiologic-pathologic correlation, Nodal staging

## Abstract

**Purpose:**

Preoperative imaging is central to cervical lymph node staging in oral squamous cell carcinoma (OSCC). However, the pathological and biological determinants underlying radiological lymph node suspicion remain poorly defined.

**Methods:**

This retrospective cohort study included 438 patients with OSCC of the tongue or floor of mouth treated with curative surgery and neck dissection between 2011 and 2021. Radiological lymph node assessment was classified using a three-level scale (unremarkable, suspicious, metastatic) and a dichotomous scale (unremarkable vs. suspicious/metastatic). Associations with patient characteristics, primary tumor features, and pathological nodal parameters were analyzed using univariable tests and multivariable ordinal logistic regression. A secondary model was performed in patients with available depth of invasion (DOI).

**Results:**

Radiological lymph node assessment was strongly associated with pathological indicators of nodal tumor burden, including number of positive lymph nodes, lymph node ratio (LNR), extranodal extension (ENE), multilevel nodal involvement, and pathological N stage (all *p* < 0.001). In multivariate analyses, LNR emerged as the strongest independent predictor of radiological lymph node category, with increasing LNR associated with higher odds of radiological suspicion. Extranodal extension remained independently associated with radiological lymph node status. Depth of invasion showed a positive association that was attenuated after adjustment for nodal tumor burden, suggesting partial mediation.

**Conclusion:**

Radiological lymph node suspicion in OSCC primarily reflects cumulative nodal tumor burden and aggressive pathological features rather than isolated nodal involvement or patient-related factors. Radiological lymph node assessment may therefore serve as a surrogate marker of biologically aggressive nodal disease in clinical decision-making.

## Introduction

Accurate preoperative assessment of cervical lymph node involvement represents a cornerstone of treatment planning in oral squamous cell carcinoma (OSCC). The presence, extent, and biological aggressiveness of nodal metastases substantially influence surgical strategy, the indication for adjuvant therapy, and ultimately patient prognosis (Kahling et al. 2016; Eskander et al. 2024). Consequently, radiological evaluation of the neck plays a vital role in guiding clinical decision-making prior to definitive surgical treatment (Vassiliou et al. 2020; Farrokhian et al. 2022).

Cross-sectional imaging modalities such as contrast-enhanced computed tomography (CT), magnetic resonance imaging (MRI), and positron emission tomography (PET) are routinely used to identify suspicious lymph nodes based on size criteria, morphological features, and contrast enhancement patterns (Wang et al. 2022; Struckmeier et al. 2023). However, radiological interpretation of cervical lymph nodes remains inherently indirect, as it relies on surrogate imaging characteristics rather than direct assessment of tumor biology (Mazzawi et al. 2018). While radiologically suspicious lymph nodes are generally associated with pathological nodal metastases, their specific correlates are increasingly recognized as an important yet incompletely defined aspect of radiological interpretation (Lorusso et al. 2025).

Previous studies on cervical lymph node imaging in OSCC have primarily focused on the diagnostic performance of preoperative staging modalities, reporting sensitivity, specificity, and predictive values of CT, MRI, and PET-CT (Mahmood et al. 2022; Struckmeier et al. 2023). Although these approaches are essential for clinical decision-making, radiological-pathological concordance remains limited, with imaging frequently underestimating nodal disease (Pakkanen et al. 2022). While individual radiological features have been linked to pathological nodal involvement, the pathological significance of radiological lymph node categories in terms of overall nodal tumor burden and biological high-risk features, such as extranodal extension (ENE), lymph node ratio (LNR) or multilevel involvement, remains incompletely characterized (Duguet-Armand et al. 2024).

In this study we sought to investigate the association between preoperative radiological lymph node interpretation and postoperative pathological tumor and nodal features. By correlating the pathological meaning of radiological lymph node suspicion, this study aims to contribute to a biologically informed framework for the interpretation of preoperative neck imaging in OSCC.

## Materials and methods

### Study design and patient selection

The study was conducted in accordance with the Declaration of Helsinki. Approval was obtained from the institutional ethics committee of the Technical University of Munich, Klinikum rechts der Isar prior to data collection (approval number: 2022-234-S-KH). Due to the retrospective nature of the study and the use of anonymized data, the requirement for informed consent was waived. This retrospective cohort study included patients treated for OSCC at our institution. All patients diagnosed with OSCC were identified via ICD-10 coding from the institutional clinical database. From this initial cohort, patients with only primary tumors of the (i) tongue or (ii) floor of the mouth were selected to reduce anatomical and lymphatic heterogeneity. Additional inclusion criteria were the availability of a documented preoperative radiological neck assessment and definitive surgical treatment with pathological evaluation of cervical lymph nodes.

**Fig. 1:**
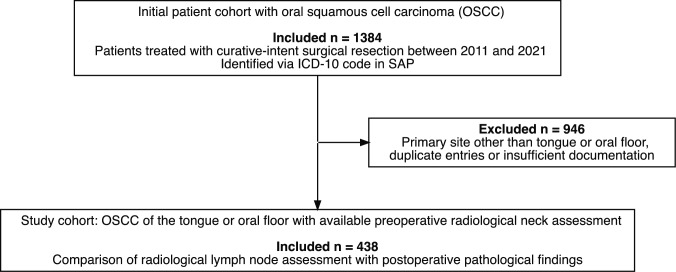
Flowchart illustrating patient selection for the study cohort

Recurrent or secondary maligniacnies were exluded. Additionally, patients were excluded if the primary tumor site was other than the tongue or oral floor, if duplicate entries were identified, another imaging modality than CT, or if clinical or pathological documentation was insufficient for analysis (Fig. 1).

### Radiological lymph node assessment

**Fig. 2:**
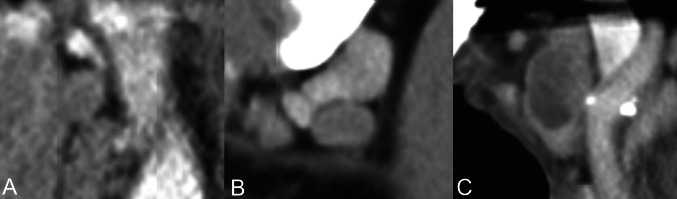
Representative radiologic assessment of cervical lymph nodes on cross-sectional imaging showing **A** an unremarkable lymph node without suspicious features, **B** a suspicious lymph node with subtle morphologic alterations suggestive of possible metastatic involvement, and **C** a metastatic lymph node with clear radiologic features of malignancy

Preoperative radiological neck assessment was performed using cross-sectional contrast enhanced CT imaging according to institutional standards. Radiological lymph node status was categorized based on the original radiology reports. Lymph nodes status was classified into three ordinal categories: radiologically unremarkable, radiologically suspicious, or radiologically metastatic (Fig. 2). For selected analyses, radiological findings were additionally dichotomized into non-suspicious versus suspicious/metastatic.

Radiological interpretation was based on standard CT criteria including lymph node size, shape, internal architecture, and signs of extranodal extension as documented in the original clinical radiology reports. Lymph nodes were considered suspicious when they showed: (1) subtle but non-definitive abnormalities, such as borderline enlargement, (2) rounded morphology, (3) focal cortical thickening, (4) partial loss of fatty hilum, (5) or mild internal heterogeneity. Lymph nodes were considered metastatic when they demonstrated more overt malignant features, such as: (1) central necrosis, (2) cystic change, (3) marked heterogeneity, (4) irregular capsular margins, or (5) radiological evidence of ENE.

### Pathological evaluation

All patients underwent surgical resection of the primary tumor with selective neck dissection. Pathological examination of resected lymph nodes was performed according to standardized histopathological protocols. The number of removed and histologically positive lymph nodes was recorded for each patient, resulting in the lymph node ration (LNR, number of positive lymph nodes divided by the total number of removed lymph nodes) (Spoerl et al. 2021). Nodal tumor burden was quantified using absolute counts of positive lymph nodes and the LNR.

Additional pathological variables included pT stage, pN stage, perineural invasion, extranodal extension, bone infiltration, and level-specific lymph node involvement.

### Variables and data collection

Demographic, clinical, radiological, and pathological variables were extracted from electronic medical records. Patient-related variables included age, sex, body mass index, ASA score (physical status classification of the american society of anesthesiologists), smoking status, alcohol consumption, and comorbidities. Tumor-related variables comprised primary tumor site, subsite localization for tongue tumors, depth of invasion, pathological tumor stage, and resection margin status.

Radiological and pathological data were merged at the patient level to allow direct comparison between preoperative imaging findings and postoperative pathological results.

### Statistical analysis

Descriptive statistics were used to summarize patient characteristics and tumor features. Categorical variables were compared using chi-square or Fisher’s exact tests as appropriate. Continuous variables were analyzed using non-parametric tests (Mann–Whitney U or Kruskal–Wallis tests) due to non-normal distributions.

Multivariate analysis was performed using ordinal logistic regression with radiological lymph node category as the dependent variable. Covariates were selected based on clinical relevance and results of univariable analyses. Model assumptions, including proportional odds, were formally assessed.

Receiver operating characteristic (ROC) curve analyses were conducted to evaluate the discriminatory ability of selected pathological variables for radiological lymph node suspicion. Statistical significance was defined as a two-sided *p*-value < 0.05. All analyses were performed using IBM SPSS Statistics Version 29.

## Results

### Study cohort and baseline characteristics

The study cohort comprised 438 patients with primary OSCC of the tongue (i) or floor of the mouth (ii) (Table 1 and Fig. 2). The median age was 62.4 (18.7–91.0) years, and 66.2% of patients were male and the mean body mass index was 24.7 ± 4.3 kg/m^2^. Most patients were classified as ASA II (74.2%), followed by ASA III (18.3%), while only a small proportion were ASA I (7.3%) or ASA IV (0.2%). Smoking was reported by 60.9% of patients, alcohol consumption by 32.0%, and diabetes mellitus by 7.5%.

**Table 1:** Patient characteristics, tumor features, radiological lymph node assessment, and pathological findings in the study cohort

**Variable**	
Age (years)	62.4 (18.7–91.0)
Sex	Male: 290 (66.2%) Female: 147 (33.6%)
Body mass index (kg/m^2^)	24.7 ± 4.3
ASA score	I: 32 (7.3%) II: 324 (74.2%) III: 80 (18.3%) IV: 1 (0.2%)
Smoking status	Yes: 266 (60.9%) No: 171 (39.1%)
Alcohol consumption	Yes: 140 (32.0%) No: 297 (68.0%)
Diabetes mellitus	Yes: 33 (7.5%) No: 404 (92.5%)
Tumor site	Floor of the mouth: 249 (56.9%) Tongue: 188 (43.1%)
Tongue subsite - Anterior third - Middle third - Posterior third - Overlapping	32 (17.0%) 95 (50.5%) 39 (20.7%) 22 (11.7%)
Radiological lymph node assessment	Unremarkable: 228 (52.0%) Suspicious: 161 (36.8%) Metastatic: 49 (11.2%)
Radiological lymph node assessment	Unremarkable: 228 (52.5%)
(dichotome)	Suspicious/metastatic: 210 (47.5%)
Number of removed lymph nodes	31 (19–95)
Positive lymph nodes (yes/no)	Yes: 153 (35%) No: 284 (65%)
Number of positive lymph nodes	0 (0–13)
Lymph node ratio	0.00 (0.00–0.67)
Extranodal extension (ENE)	Yes: 59 (13.5%) No: 378 (86.5%)
Lymph node level metastasis - Level Ia - Level Ib - Level II/III	*Percentages refer to the total study cohort* 33 (7.5%) 62 (14.2%) 149 (34.1%)
Depth of invasion, DOI (mm)	6.0 (0–27)
Bone infiltration	Yes: 64 (14.7%) No: 371 (85.3%)
Pathological T stage	pT1: 180 (41.1%) pT2: 131 (30.1%) pT3: 63 (14.4%) pT4: 63 (14.4%)

Primary OSCC were located in the floor of the mouth in 56.9% and in the tongue in 43.1% of cases. Among tongue carcinomas, tumors were most frequently located in the middle third (50.5%), followed by the posterior third (20.7%) and anterior third (17.0%); overlapping subsites were observed in 11.7%. The median depth of invasion was 6.0 mm (0–27 mm), and bone infiltration was present in 14.7% of cases. Early pathological tumor stages (pT1–pT2) accounted for 71.2% of patients, while advanced tumors (pT3–pT4) comprised 28.8%.

Radiological lymph node assessment classified 51.9% of patients as unremarkable, 36.8% as suspicious, and 11.2% as metastatic; dichotomized assessment identified 47.5% of patients as radiologically suspicious or metastatic. Histopathological analysis revealed a median of 31 removed lymph nodes (19–95). Most patients had no nodal metastases, resulting in a median of 0 positive lymph nodes (0–13) and a median LNR of 0.00 (0.00–0.67). Extranodal extension was present in 13.5% of patients. With regard to nodal distribution, metastases most frequently involved levels II/III (34.1%), followed by level Ib (14.2%) and level Ia (7.5%), with percentages referring to the total study cohort.

### Univariate associations between radiology and clinicopathological variables

In univariate logistic regression analyses, radiological lymph node status showed strong and consistent associations with pathological indicators of nodal tumor burden and disease aggressiveness (Table 2). Increasing radiological suspicion was significantly associated with ENE, the presence and number of histologically positive lymph nodes, higher LNR, multilevel nodal involvement, and advanced pN stage in both the three-level and dichotomous assessments (each *p* < 0.001, respectively). Similarly, radiological lymph node status was significantly associated with pT stage and depth of invasion, with stronger effects observed in the dichotomous assessment (*p* < 0.001). Bone infiltration was also significantly associated with radiological lymph node categories for the three-level assessment (*p* < 0.001) and for the dichotomous assessment *(p* = 0.002).

**Table 2:** Univariable analysis of associations between patient characteristics, tumor features, and pathological findings with radiological lymph node assessment using a three-level classification (unremarkable, suspicious, metastatic) and a dichotomous classification (unremarkable vs. suspicious/metastatic)

Category	Variable	p-value (3-level assessment)	p-value (dichotomous assessment)
Patient characteristics	Age	0.176	0.220
	Body height	0.135	0.199
	Body weight	0.324	0.681
	Body mass index (BMI)	0.906	0.572
	ASA score	0.040*	0.124
Risk factors	Smoking	0.067	0.089
	Alcohol consumption	0.213	0.354
	Diabetes mellitus	0.768	0.630
Primary tumor – extent	Pathological T stage	< 0.001*	< 0.001*
	Depth of invasion (DOI, mm)	0.001*	< 0.001*
	Bone infiltration	< 0.001*	0.002*
	Tumor location (tongue vs. floor of mouth)	0.926	0.727
	Tongue subsite	0.008*	0.280
Nodal tumor burden	Number of removed lymph nodes	< 0.001*	< 0.001*
	Number of positive lymph nodes	< 0.001*	< 0.001*
	Lymph node ratio	< 0.001*	< 0.001*
	Pathological N stage	< 0.001*	< 0.001*
Biological high-risk features	Extranodal extension (ENE)	< 0.001*	< 0.001*
	Multilevel metastasis	< 0.001*	< 0.001*
	Perineural invasion (Pn stage)	< 0.001*	< 0.001*

P-values < 0.05 were considered statistically significant and are marked with an asterisk (*)

With regard to primary tumor characteristics, tongue subsite was significantly associated with radiological lymph node status in the three-level assessment (*p* = 0.008), but this association was not retained after dichotomization. In contrast, overall tumor location (tongue versus floor of the mouth) showed no association with radiological lymph node status in either analysis (*p* = 0.727*)*.

Patient-related factors were not consistently associated with radiological lymph node assessment. Age, body height, body weight, body mass index, smoking status, alcohol consumption, and diabetes mellitus showed no significant associations in either the three-level or dichotomous analyses. The ASA score demonstrated an association with radiological lymph node status in the three-level assessment (*p* = 0.040), but this effect was not confirmed in the dichotomous analysis.

### Multivariate regression analysis

Multivariate logistic regression analysis was performed with radiological lymph node status (unremarkable, suspicious, metastatic) as the dependent variable (Table 3). In the primary model including the full cohort (n = 434), lymph node ratio categories, ENE (*p* = 0.019), and bone infiltration (*p* = 0.048) emerged as independent predictors of higher radiological lymph node categories, while age was not independently associated.

**Table 3:** Multivariate logistic regression analyses of factors associated with radiological lymph node assessment

Variable	OR	95% CI	*p*-value
Age (per Year)	0.99	0.97–1.01	0.17
Bone infiltration (yes vs. no)	**1.7**	1.01–3.0	**0.048**
LNR 0–0.1 vs. 0	**4.8**	3.0–7.8	** < 0.001**
LNR > 0.1 vs. 0	**10.0**	4.0–25.0	** < 0.001**
ENE (yes vs. no)	**2.2**	1.1–4.2	**0.019**

Results are presented as odds ratios (ORs) with 95% confidence intervals (CIs) and p-values < 0.05 were considered statistically significant. (LNR = lymph node ratio; ENE = extranodal extension)

**Fig. 3:**
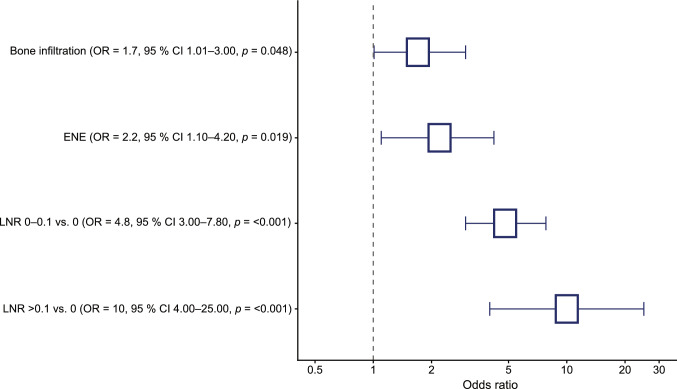
Forest plot of the multivariate logistic regression model. Squares represent odds ratios (ORs), horizontal lines indicate 95% confidence intervals (CIs), and the dashed vertical line denotes the null effect (OR = 1). Odds ratios are displayed on a logarithmic scale. Only statistically significant variables are shown. (vs. = versus; LNR = lymph node ratio; ENE = extranodal extension)

Compared with patients without nodal involvement (LNR = 0), patients with a LNR between 0 and 0.1 had significantly increased odds of being classified into higher radiological categories (OR ≈ 4.8, *p* < 0.001, Fig. 3). This effect was even more pronounced in patients with a LNR greater than 0.1 (OR ≈ 10.0, *p* < 0.001). Extranodal extension remained independently associated with higher radiological lymph node categories (OR ≈ 2.2, *p* = 0.019), as did bone infiltration (OR ≈ 1.7, *p* = 0.048). Age was not independently associated with radiological lymph node status.

Model fit was good, with no evidence of lack of fit on Pearson or deviance testing, and the proportional odds assumption was met (parallel lines test *p* > 0.05).

### Sensitivity analysis including depth of invasion

In a secondary multivariate ordinal logistic regression model restricted to patients with documented depth of invasion (n = 141), LNR categories remained the strongest independent predictors of radiological lymph node status (Table 4). Compared with patients without nodal metastases, those with a LNR of 0–0.1 and ≥ 0.1 showed significantly increased odds of being classified into higher radiological lymph node categories (each *p* < 0.001, respectively).

**Table 4:** Multivariate logistic regression analysis of factors associated with radiological lymph node status in patients with documented depth of invasion (n = 141)

Variable	OR	95% CI	p-value
Age (per year)	0.98–0.99	~ 0.95–1.01	0.20
Depth of invasion (per mm)	1.07	1.01–1.14	**0.03**
Bone infiltration (yes vs. no)	0.27–0.35*	0.06–1.02	0.052
LNR 0–0.1 vs. 0	1.9	1.1–2.8	** < 0.001**
LNR ≥ 0.1 vs. 0	4.8	2.9–6.8	** < 0.001**

Results are presented as odds ratios (ORs) with 95% confidence intervals (CIs). Lymph node ratio (LNR) was entered as a categorical variable with “0” as the reference category. P-values < 0.05 were considered statistically significant

Depth of invasion was independently associated with radiological lymph node status, with increasing DOI corresponding to higher radiological suspicion (OR 1.07 per mm, 95% CI 1.01–1.14; *p* = 0.03). Bone infiltration demonstrated a borderline association with radiological lymph node categories, which did not reach conventional levels of statistical significance after adjustment for nodal tumor burden (*p* = 0.052). Age was not independently associated with radiological lymph node status in this model (*p* = 0.20).

### Receiver operating characteristic analysis

**Fig. 4:**
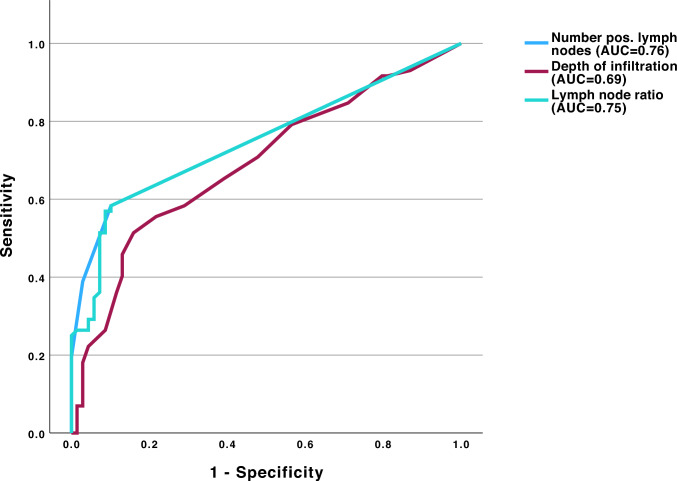
Receiver operating characteristic (ROC) curves illustrating the ability of pathological parameters to discriminate radiologically suspicious lymph node findings. The number of positive lymph nodes (AUC = 0.76) and the lymph node ratio (AUC = 0.75) showed good discriminatory performance, while depth of invasion demonstrated moderate accuracy (AUC = 0.69). Sensitivity is plotted against 1 − specificity

Receiver operating characteristic (ROC) analyses were performed using radiological suspicion (unremarkable vs. suspicious/metastatic) as a binary outcome (Fig. 4). Depth of invasion demonstrated acceptable discriminatory ability (AUC 0.69, 95% CI 0.60–0.78), while the number of positive lymph nodes (AUC 0.76, 95% CI 0.67–0.84) and LNR (AUC 0.75, 95% CI 0.67–0.83) showed good discrimination. A DOI threshold of approximately 8–9 mm (Youden´s index 0.354) provided the optimal balance between sensitivity and specificity for radiological lymph node suspicion. Identified thresholds were derived for descriptive purposes only and were not used to define radiological categories.

### Analysis of radiological false-negative cases

Among patients classified as radiologically unremarkable, a small subset of 9 patients (2.1%) exhibited high-risk pathological features, defined as the presence of ENE or a high LNR, and were therefore considered radiological false-negative cases. Seven patients had pT1 tumors and two had pT2 tumors with a median DOI of 3 (1–9) mm. Tumor location was evenly distributed, with four cases arising in the tongue and five in the oral floor. Due to the small number of false-negative cases and limited variability within this subgroup, further statistical comparisons were not performed.

## Discussion

In this study, we systematically examined what radiological lymph node assessment represents in terms of underlying pathological tumor burden and biological high-risk features in only primary OSCC of (i) the tongue and (ii) the floor of the mouth. While previous work has largely focused on diagnostic accuracy metrics such as sensitivity and specificity of imaging for nodal staging, considerably less attention has been paid to the pathological correlates that drive radiological lymph node suspicion (Struckmeier et al. 2023). Our findings indicate that radiological lymph node assessment primarily reflects the extent of nodal tumor burden and associated high-risk pathological features, whereas patient-related characteristics play a negligible role. This concept is in line with recent radiology-pathology concordance studies suggesting that radiological nodal positivity preferentially identifies advanced nodal disease rather than isolated microscopic metastases (Arun et al. 2021; Duguet-Armand et al. 2024).

### High-risk pathological features

Across both uni- and multivariate analyses, measures of nodal tumor burden, most prominently the LNR, emerged as the strongest and most consistent predictors of radiological lymph node classification. Compared with patients without nodal involvement (LNR = 0), patients with a LNR between 0 and 0.1 had significantly increased odds of being classified into higher radiological categories (OR ≈ 4.8, *p* < 0.001, Fig. 2). This effect was even more pronounced in patients with a LNR greater than 0.1 (OR ≈ 10.0, *p* < 0.001), indicating a strong dose–response relationship between nodal tumor burden and radiological assessment. Although LNR and number of metastatic lymph nodes are well-established prognostic markers in OSCC (Kang et al. 2022; Tsai et al. 2022; Struckmeier et al. 2024; Dudkiewicz et al. 2025), their relationship to radiological lymph node assessment has rarely been examined. Our findings extend prior observations by demonstrating that radiological interpretation appears to reflect cumulative nodal tumor burden rather than the mere presence of nodal metastasis. This observation aligns with reports showing that radiological sensitivity increases substantially with larger nodal size, multiple involved nodes, or advanced nodal disease, while remaining limited for small-volume or morphologically subtle metastases (Arun et al. 2021; Duguet-Armand et al. 2024).

Extranodal extension showed a robust and independent association with radiological lymph node assessment. This finding is biologically plausible, as pathological ENE is characterized by capsular disruption and infiltration of surrounding tissues, features that translate into irregular nodal margins and soft-tissue extension detectable on cross-sectional imaging. Prior studies have demonstrated that radiological detection of ENE is highly specific but only modestly sensitive, particularly identifying major rather than minor ENE (Henson et al. 2024). Importantly, pathological ENE has been consistently linked to higher lymph node ratio, increased nodal burden, and adverse oncologic outcomes (Ho et al. 2017; Huang et al. 2019). Our results therefore support the concept that radiological lymph node suspicion captures biologically aggressive nodal disease with direct prognostic and therapeutic relevance rather than serving as a purely anatomical descriptor. Nevertheless, a small subset of nine patients (2.1%) exhibited high-risk pathological features (ENE or a high LNR) and were therefore considered radiological false-negative cases; these patients predominantly had early-stage primary tumors (n = 7 pT1, n = 2 pT2) with a low median depth of invasion of 3 mm (range 1–9 mm), and showed no clear predominance with regard to tumor location (tongue n = 4, floor of mouth n = 5). Although limited by the small sample size, this pattern suggests that radiological assessment may be most prone to miss biologically aggressive nodal disease in early-stage tumors with low depth of invasion.

Primary tumor-related factors demonstrated a more subtle relationship with radiological lymph node findings. Depth of invasion was significantly associated with radiological lymph node status in univariate analyses and remained positively associated in adjusted models restricted to patients with available DOI data (*p* = 0.03).However, this effect was attenuated after adjustment for nodal tumor burden, suggesting partial mediation through the development of more extensive nodal disease. This interpretation is consistent with prior studies identifying DOI as a key driver of nodal metastasis risk rather than an independent determinant of radiological nodal appearance (Alqutub et al. 2024; Mrosk et al. 2024). Receiver operating characteristic analysis identified an exploratory DOI threshold of approximately 8–9 mm for radiological lymph node suspicion. This value lies between the 5 mm and 10 mm cutoffs used in the AJCC 8th edition for T-stage upstaging and may therefore be biologically plausible rather than contradictory (Amin et al. 2017). While the AJCC thresholds are intended to reflect the risk of nodal metastasis, our finding relates specifically to the likelihood that nodal disease becomes radiologically apparent. It is conceivable that metastatic potential increases beyond 5 mm DOI, whereas radiological lymph node suspicion becomes more evident only at greater depths of invasion, when cumulative nodal tumor burden and aggressive nodal features are more likely to be present.

Notably, most patient-specific characteristics, including age, body composition, smoking status, alcohol consumption, and diabetes mellitus, were not independently associated with radiological lymph node assessment. The ASA score showed a weak and inconsistent association in univariable analyses, which did not persist across alternative radiological classifications. Overall, while patient-related factors may influence general prognosis and treatment tolerance (Budach and Tinhofer 2019), their direct impact on radiological nodal appearance appears limited compared with tumor-specific and nodal biological features.

### Persepectives

Our findings help contextualize the well-documented limitations of imaging in nodal staging. Radiological assessment is known to underestimate microscopic nodal metastases, particularly in early-stage disease, due to limited sensitivity for small or morphologically normal nodes (Mazzawi et al. 2018, Alan et al. 2026). However, the present results indicate that radiological lymph node positivity preferentially identifies patients with higher nodal tumor burden, ENE and multilevel nodal involvement, features that are strongly associated with adverse oncologic outcomes (Dolens et al. 2021; Mamic et al. 2021). In this context, radiological lymph node assessment may be better understood as a surrogate marker of biologically aggressive nodal disease rather than a binary detector of nodal metastasis.

In addition to conventional histopathological parameters, including tumor size, DOI, anatomical site, number of positive lymph nodes, and LNR (Ettl et al. 2014; Rempel et al. 2018), comprehensive immunohistochemical profiling (e.g., PD-L1, PLOD1, Desmoglein-3, ALDH1, and Notch1) (Götz et al. 2018; de Freitas Filho et al. 2021; Sun et al. 2021; Troeltzsch et al. 2022) should be respected in the future. These molecular and histopathological features could be integrated into the articifial inteligent framework as part of a multidimensional data architecture, thereby enriching the predictive model beyond imaging characteristics alone. Ultimately, the integration of imaging, histopathology, and molecular data into a validated articifial inteligent framework may contribute to more precise, risk-adapted decision-making in neck management. Such an approach holds the potential to support surgical de-escalation strategies while maintaining oncologic safety, particularly in early-stage, clinically node-negative OSCC.

### Limitations

Several limitations must be considered. The retrospective design introduces inherent selection bias, and depth of invasion was not available for all patients, necessitating a secondary model restricted to a subset of the cohort. Radiological assessments were based on routine clinical reports rather than centralized re-review, introducing potential interobserver variability but reflecting real-world clinical practice. Despite these limitations, this study benefits from a large, well-characterized cohort, detailed pathological nodal assessment, and the application of both three-level and dichotomous radiological classifications. By integrating quantitative nodal measures such as lymph node ratio with established biological risk factors, our analysis provides a detailed understanding of what radiological lymph node suspicion represents in clinical practice and highlights its role as an indicator of cumulative nodal tumor burden.

## Conclusion

In conclusion, radiological lymph node assessment in OSCC primarily reflects underlying nodal tumor burden and biologically aggressive disease features, particularly lymph node ratio and extranodal extension. Local tumor aggressiveness contributes to radiological findings but appears to exert its influence largely through the development of extensive nodal disease. These insights support the use of radiological lymph node assessment as a marker of nodal disease severity rather than solely as a diagnostic tool for nodal metastasis detection and may help refine risk stratification and treatment planning in patients with OSCC.

## Data Availability

The datasets generated during and analysed during the current study are available from the corresponding author on reasonable request.

## Abbreviations

AI: Artificial intelligence
ASA: American Society of Anesthesiologists (physical status classification)
AUC: Area under the curve
BMI: Body mass index
CI: Confidence interval
CT: Computed tomography
DOI: Depth of invasion
ENE: Extranodal extension
LNR: Lymph node ratio
MRI: Magnetic resonance imaging
OR: Odds ratio
OSCC: Oral squamous cell carcinoma
PET: Positron emission tomography
pN: Pathological nodal stage
pT: Pathological tumor stage
ROC: Receiver operating characteristic
